# Hospital of Nantes

**Published:** 1829-04-01

**Authors:** 


### HOSPITAL OF NANTES.

CLINICAL REPORT; by A. LAENNEC, M.D. Physician of the Hôtel Dieu of Nantes.

The Hospital of Nantes is capable of holding 500 patients, and presents a good field for observation. Dr. Laennec appears to be a zealous and intelligent practitioner, and we have much pleasure in the introduction of a review of one of his recent clinical reports from the above institution.

1. ANGINA PELLICULARIS. This appellation has been lately given to those inflammations about the throat, in which exudations, or false membranes, are thrown out, during the phlogosis, on the internal surfaces of the air-passages, pharynx, &c. Our readers are acquainted with M. Bretonneau's writings and practice in this class of complaints. Dr. Laennec has been very successful, by means of the insufflation of finely powdered alum in these pellicular anginæ—and, even in the common anginæ tonsillares, he considers the aforesaid application as capable of subduing the tumefaction of the tonsils and neighbouring parts with a greater degree of rapidity than any other remedy. It is well known that, in some cases of small pox, there is an inflammation propagated to the larynx and trachea, which is often the cause of the fatal termination. Since our author has employed the aluminous insufflations, he has seen no fatal result of this variolous angina. In one case of œdema of the glottis, a very dangerous disease, the aluminous application produced very quick relief. We shall give an outline of this case.

*Œdema of the Glottis.* Peter Burgoin, aged 46 years, entered the Hôtel Dieu of Nantes on the 29th of November. Four days previously he had been exposed to a current of cold air, while in a state of perspiration, soon after which he was seized with sore throat and difficulty of swallowing, followed by a sense of burning heat along the trachea, and constant cough. The oppression, difficulty of breathing, glairy expectoration, &c. increased, and when he entered the hospital, he was in a very dangerous condition—each inspiration being effected with great labour. His face was of a violet colour—pulse hard and full. The alum insufflation employed twice. It caused some irritation at first, and much cough during which he inhaled with considerable difficulty. Afterwards the breathing became more easy—he was nearly seven hours without coughing, and the night was passed in comparative tranquillity. The insufflation was practised every day till the disease yielded. Very few other medicines were employed—none of any efficiency. Expectoration came on about the fourth day after he was received, and then the symptoms became much mitigated.

II. Encysted Tumour of the Right Ovarium cured.—Julia M. aged 26 years, not married, of robust constitution, and who had always enjoyed good health, became pregnant at the age of 22, and was delivered without any accident at the full period. After this she remained in good health till January 1826, when, without any apparent cause, she became affected with a sense of weight in the abdomen; and soon afterwards perceived a round tumour in the right iliac fossa, capable of being moved about by the hand. The tumour remained stationary till the month of April, when the abdomen began to progressively enlarge, and, in another month, was very inconvenient and uneasy. The patient entered the Hôtel Dieu on the 29th of May. The abdomen was then found to be tense and distended—and a pyriform tumour was very distinctly felt in the right iliac and hypochondriac regions, apparently descending into the pelvis, with a diminished diameter. There was a slight sense of fluctuation in the abdomen. Auscultation discovered no particular noise or pulsation. The patient was somewhat emaciated; but her appetite was good, tongue clean, and all the other functions natural, even the menstruation. The state of the uterus was examined, and found to be natural. The diagnosis was a matter of some difficulty. Was this a case of extra-uterine fœtation—or of hydatids in the ovary? Dr. L. determined to wait a little before he decided. During the month of June, the tumour augmented in size, especially that part which appeared to descend into the pelvis. Towards the end of the month, the patient complained of shooting pains in that part of the abdomen, rendering walking exercise very distressing. The functions, however, continued healthy, and she menstruated on the 1st of July. Our author now became convinced that the disease was a case of ovarian hydatids, and he determined on the use of chlorine baths. The patient was put into one every second day. In each bath was dissolved five pounds of hydrochlorate of soda. After the sixth bath, which was on the 17th of July, the size of the abdomen rapidly augmented—and the tumour ceased to be so definitely bounded as before. In a few more days, the abdomen became as large as that of a female in the ninth month of pregnancy. The tumour was evidently in the right side of the abdomen; but its boundaries were very indefinite. On the 29th of July, the patient, on coming out of the twelfth bath, complained of violent cephalalgia. In the same evening she had a rigor, followed by copious perspiration. The abdomen now became uniformly soft and more enlarged in all directions. It was void of tenderness on pressure. The patient complained of excessive debility. The excretion of urine became notably diminished. 30th. The abdomen was found to be extremely soft, and void of tenderness. No tumour could now be felt—but Dr. L. says that he thought he could feel a kind of *half empty cyst* or bag in the right side of the abdomen—a feeling which might be correct, but which we suspect to be somewhat of the *pre-conception.* The abdomen was sustained by a proper bandage; and the baths were continued, with the muriate of soda. On the *9th of August,* it was found that the abdomen had become very much smaller. Fluctuation was very indistinct—the appetite good—all the functions regular. On this day the patient was seized with a natural—or, at all events, a non-mercurial salivation, so abundant that she discharged four large cups of saliva in 24 hours. *10th August.* The salivation is less copious, but still continues. From this period the abdomen daily diminished in size. On the 12th of this month, she took her nineteenth and last chlorine bath—her convalescence was rapid—and on the 19th August, she was discharged from the hospital completely cured.

*Remarks.* The foregoing case is a very interesting one—and we hope soon to be able to add, from private practice, another of considerable interest. It appears that Dr. Laennec was induced to prescribe the chlorine baths from the recommendation of his cousin (the great Laennec) who had employed them in the case of sheep affected with hydatids. Whatever share the baths may have had in the cure of this complaint, we are inclined to agree with the author that the above was a case of hydatids—that the mother hydatid had burst—and that absorption of the effused fluid had happily taken place.

III. Trismus—Opisthotonos, from Cold—cure.—Peter Dupavé, aged 27 years, was carried to the Hôtel Dieu of Nantes, on the 12th of June. Twelve days previously, he went into cold water, being then in a state of perspiration. He was seized with a rigor, and three days afterwards he experienced a difficulty in moving the lower jaw. This difficulty daily increased—the muscles of the spine became rigid, with excessive pain along the vertebral column. He had been bled from the arm, and leeches had been applied to the anus, without any relief. When admitted into hospital, on the 12th of June, there was complete trismus—deglutition very difficult—head drawn backwards—in short, all the symptoms of opisthotonos. Constipation was obstinate—pulse quick—no sleep. *Four grains of opium.* Next day, 13th June, our author saw the patient, and prescribed 12 grains of emetic tartar in a vegetable infusion, a table spoonful every two hours —a large detraction of blood from the arm. The above quantity was taken in 24 hours, and produced no effect on stomach or bowels. Slept some in the night. 14th, The trismus rather less rigid—*the same prescription—venesection.* *15th. The same medicine—no bleeding.* The trismus and opisthotonos more reduced. The antimonial medicine was continued for some days longer—and strong purgatives were necessary, to obviate obstinate constipation. The patient was discharged cured on the 7th of July.

IV. General Phlebitis—or Purulent Concretions in Veins.—The following case will prove a very valuable addition to the mass of facts brought forward by Mr. Arnott on phlebitis and its consequences.

*Case.* F. Gibeau, aged 28 years, was delivered of twins on the 8th July, 1826. On the 9th and 10th she went on well, though the lochia were scanty. On the 11th her breasts swelled and became painful, the pain extending to the axillæ, and along the left side of the chest, with rigors, fever, some delirium, diarrhœa, and vomiting. Pains now seized various other articulations, and she was brought to the hospital on the 14th July. Her countenance was now pallid, features shrunk, tongue coated, dry, and red; thirst, abdomen tense and tender on pressure, a large umbilical hernia easily reducible, diarrhœa, vomiting, quick pulse, cough, oppression, knee-joints red, swelled, and painful—œdema of the lower extremities, which were tense, shining, white, elastic, and did not leave pits on pressure. The right mamma was swelled and shining. The diagnosis was —"active œdema of puerperal women" —"*œdeme actif des nouvelles accouchées*: in other words, phlegmatia dolens. Twenty ounces of blood were taken from the arm—lowest scale of regimen—emollient cataplasms to the breast and abdomen. The venesection was repeated the next two days, the blood being much inflamed. *The left arm became the seat of great pain.* 19th July. There are two gangrenous spots on the right mamma—a similar appearance was presented on the labia pudendi. The left arm shewed the same kind of œdematous swelling as the lower extremities. The phlegmasia, which was thus extended to so many organs and parts, was now treated by the tartrite of antimony, in doses of six grains per diem. This plan was pursued during six days—from the 20th to the 26th July, in the course of which time, the diarrhœa ceased; but the œdematous swellings remained. The diarrhœa returned on the 30th July, and continued till the patient's death, which took place on the 15th of August.

*Dissection.* We must pass over a great many minute particulars, in order to preserve the more important parts of the dissection. There were some points of hepatization in the lungs, but no purulent depôts. The pharynx and œsophagus were completely lined with an exudation resembling soft morter, forming a tube that reached to the cardia, and there terminated. The mucous membrane of the œsophagus itself was pale when denuded. The peritoneum was healthy, as was the mucous membrane of the stomach and intestines, except some small portions of the colon. The uterus was the size of a fist, and its cavity filled with a sanguineo-purulent fluid. The parietes of the organ were livid—and, towards the fundus, in a state of mollescence. There was a vast depôt of suppuration about the left shoulder joint—but the other articulations, so swelled and inflamed during life, were now collapsed and colourless. The principal veins of the left arm were filled with a white tubular concretion, non-adherent to the internal surfaces of the vessels. The tubular concretion itself was filled with pus exactly similar to that found around the joint. The internal coats of the vein were remarkably pale. The iliac veins contained polypous concretions not at all analogous to those above described. The hypogastric and femoral veins, however, presented tubular concretions filled with pus, down even to the ankle. Finally, the pulmonary *arteries* presented the same appearances as those described in the veins.

*Remarks.* When we consider the state of the internal surfaces of the vessels in which these concretions filled with pus, were found,—pale and non-adherent to the said concretions—we must conclude with the author that the phenomena cannot be legitimately traced to phlebitis. "I am rather disposed, says he, to believe that this concretion was formed at the expense of the blood itself—and that it was the consequence of a mixture of pus, that is, an inorganic fluid, with one endowed with life." In whatever way the facts may be viewed, they tend to support the doctrine of the humoral pathology, so long and so unjustly ridiculed by sciolists and visionary solidists.—Revue Medicale.

